# Parents’ experiences of paediatric palliative care in the community healthcare system: a qualitative study

**DOI:** 10.1177/26323524231193036

**Published:** 2023-08-28

**Authors:** Stine Andreassen Rud, Eirin Skagestad, May Aasebø Hauken

**Affiliations:** Center for Crisis Psychology, Faculty of Psychology, University of Bergen, Bergen, Norway; Center for Crisis Psychology, Faculty of Psychology, University of Bergen, Bergen, Norway; Center for Crisis Psychology, Faculty of Psychology, University of Bergen. Møllendalsbakken 9, Postbox 7807, Bergen, 5020, Norway

**Keywords:** community healthcare, home care, paediatrics, palliation, parents’ experiences, qualitative study

## Abstract

**Background:**

Having a child with a life-limiting illness is a situation that is relatively rare and represents a multidimensional burden on the family. Paediatric palliative care (PPC) aims to maintain the quality of life for the ill child and the family. Traditionally, most PPC has been provided at a specialist healthcare level, but research indicates that most families wish to spend as much time at home as possible. However, we have limited knowledge of PPC in community healthcare, especially from the parent’s perspective. This knowledge is important to provide optimal home-based PPC.

**Objectives:**

To explore parents’ experiences of PPC within the community healthcare system.

**Design:**

Qualitative study with an interpretive descriptive design.

**Methods:**

In all, 11 parents of children with different life-limiting illnesses were interviewed after the child’s death using a semi-structured interview guideline. Data were analysed using systematic text condensation. Consolidated criteria for reporting qualitative research (QOREQ) was followed.

**Results:**

The parents’ experiences were captured in five main themes: (i) ‘Interaction with hospital and community services’, (ii) ‘Parents did not always get the help they needed’, (iii) ‘The child’s needs became increasingly complex’, (iv) ‘When the end came’ and (v) ‘The parents asked for an ordinary life in an unordinary situation’. Each main theme was further elaborated by two subthemes.

**Conclusion:**

Overall, the parents experienced PPC in the community as limited and fragile, and as lacking flexibility, coordination and professional competence related to the children’s complex needs. There appears to be potential for improvement in PPC through improved care coordination between the hospital and the community healthcare services, involving the community healthcare system at an early timepoint in the illness trajectory, including a family focus, and providing accessibility, flexibility and care coordination of community services.

**Registration and reporting guidelines:**

The study is registered in the institutional system for research project (RETTE; ID number F2082).

## Introduction

Worldwide, children (<18 years of age) comprise 35% of the global population and the number of children living with a life-limiting condition and in need of paediatric palliative care (PPC) is increasing and estimated to be as high as 21 million.^1^ Children constitute 21% of the Norwegian population and it is estimated that more than 3800 children need paediatric palliation, but no official documentation is available. Of Norwegian children in need of PPC, approximately 40% have a neuromuscular disease, 40% have a genetic condition and 20% have cancer.^2^

PPC is defined by the World Health Organization (WHO) as

the active and total care of the child’s body, mind, and spirit, which also involves giving support to the family. It begins when illness is diagnosed and continues regardless of whether a child receives treatment directed at the disease or not.^3^

Consequently, PPC must be adapted to individual needs, to increase the quality of life for both the children and their families. The WHO guidelines also emphasize the importance of efficient multidisciplinary collaboration, including family and community resources.

The definition of PPC aligns with the definition of adult palliative care, but there are several distinct differences. The PPC patient population is relatively small and is characterized by frequent ambiguity regarding the child’s prognosis, life expectancy and functional outcome. The care must fit the child’s continual physical, cognitive, emotional, and social development.^1^ Furthermore, the child’s illness impacts the entire family, and usually increases parental care burden. Children’s medical symptoms may be more challenging to discover and treat than those of adults. All these factors underline the complexity of PPC and the need for specific competence within the healthcare system.^4^

PPC can be provided at hospitals, hospices and community health centres, and in the family home.^3^ However, access to PPC has mainly been provided through hospitals and specialist health services, both internationally and nationally.^1,2^ Consequently, most research has been performed in this context. In contrast to this practice, studies document that families prefer to spend the final phase of their child’s life in the family home, with the support of specialist palliative care professionals.^5^ The European Association for Children in Hospital^6^ states that children shall be admitted to hospital only if they cannot be equally well provided for at home. In Norway, hospitals are organized at the specialist healthcare level by four Regional Health Authorities. Primary health care is organized by 356 municipalities. Nationally, the emphasis on PPC development is quite new, especially in primary healthcare. However, in a new national guideline (2017), based on WHO’s definition and international standards, it is stated that PPC for children with a life-limiting disease or disorder should be initiated at the time of diagnosis, and that PPC primarily should be provided in the home.^2,7^ To strengthen the quality of PPC both within the hospitals and in the municipalities, each Regional Health Authority established multidisciplinary paediatric palliative teams at regional hospitals and within paediatric departments at local hospitals.^2^ Research shows that home care presents an opportunity for parents to take care of their child in a less stressful environment, keeping the family together, strengthening family life, maintaining the daily activities of siblings and involving social support from extended family and friends.^8^ Neilson *et al.*^9^ also found that providing PPC at home can result in the child feeling happier, and parents coping better with bereavement.

Although caring for the child at home has its benefits, it can also be physically and emotionally exhausting for the parents to provide medical care and emotional support for a child with a life-limiting illness.^10^ The perceived quality of PPC affects parental well-being after the child’s death, and bereaved parents and siblings are at risk of complicated grief trajectories, as well as post-traumatic stress disorder PTSD.^11,12^ One example is that insufficient symptom management has been linked to lower quality of life in paediatric patients and long-term parental grief and distress.^13,14^

The existing research indicates that symptom management, emotional and psychological aspects of care and efficient care coordination are the most important factors for successful home-based PPC.^15–17^ Access to respite has been emphasized as an important factor in parental coping in PPC.^5,18^ Lack of proper respite may impact the quality of the time the parents spend with their children.^19^ Another important factor is a trusting relationship between the family, the care professionals and the hospital.^5^ Distrust could result in parental anxiety and a feeling of overwhelming responsibility.^8^

From the perspective of community healthcare systems and providers, several challenges are raised. First, PPC represents a very small population, so palliative units in each municipality may be very experienced in adult palliation but have limited experience and competence in PPC. Several studies state that palliative healthcare professionals without specific competence in PPC find it challenging to adapt the adult standard of palliation to fit the needs of children and their families, or to adjust the procedures and coordinate the different services involved in caring for the child.^20,21^ Second, even if the families stay at home, the research indicates that they mostly rely on PPC services provided by the specialist healthcare level.^2^ Consequently, limited knowledge exists of parents’ own experiences of important factors for PPC provided by community healthcare.

## Study aim

This study is part of a larger project that aims to determine the essential factors for optimal paediatric palliation in the community. This knowledge is essential for the development of evidence-based models for community PPC. Consequently, the aim of the current study was to give a voice to parents who had experienced losing a child from a serious disease and their experiences of PPC provided by the community system.

## Method

To answer the research question, a qualitative study with an interpretive descriptive design was conducted.^22^ This design is characterized by the creation of new insight relevant to clinical practice through a constructivist and naturalistic orientation based on the informants’ lived experiences and the researchers’ interpretations.^22,23^ This approach is considered appropriate for the investigation of complex issues that last over time, as is the case in this study.

### Ethics and consent to participate

The study was assessed by the Regional Committee for Ethics in Medical and Health Research (REK) and was concluded to be outside the Norwegian Act on Medical and Health Research (reference number: 435941). The project was approved by the Norwegian Data Protection Authority (reference number: 270888) and registered in the institutional system for research project (RETTE; ID number F2082).

The participants received verbal and written information about the study, including statements of voluntary participation and the possibility to withdraw at any time. Participants gave their telephone number or e-mail address to the researcher and were then provided with additional information about the interview process. All participants re-confirmed their informed consent verbally on tape before the interviews started. All participants were allowed to contact the researcher or a cancer coordinator post-interview for follow-up, but none requested this. The researchers followed established guidelines for the preservation of anonymity and the safe handling of the data based on the institutional routines and the Declaration of Helsinki.^25^

### Recruitment and inclusion criteria

As this is a hard population to reach, participants were recruited through a convenience sampling procedure.^23^ Invitations to participate were issued through the Norwegian Childhood Cancer Society and LionMums, as well as by cancer coordinators in the municipal healthcare system. Eligibility was based on the following inclusion criteria: (a) Parents, who have experienced, (b) the loss of a child, with (c) severe illness. In all, 13 participants were recruited, but two withdrew prior to the interview phase. Consequently, 11 participants were included – 10 females and 1 male (one couple). The duration of the children’s illness varied from 8 months to 15 years. Mean time since the death of the child was nearly 5 years. Informant data are provided in Table 1.

**Table 1.** Participants’ sociodemographic and medical data (*N* = 11).

Variable	*N* (%)	Median (SD/range)
Gender		
Female	10 (90.9)	
Male	1 (9.1)	
Age		47.0 (5.1/37–54)
Civil status		
Married/cohabiting	8 (72.7)	
Single/divorced	3 (27.3)	
Highest education		
Senior high school	1 (9.1)	
University/university college	10 (90.9)	
Deceased child		
Gender		
Male	10 (90.9)	
Female	1 (9.1)	
Diagnosis		
Cancer	5 (45.5)	
Congenital disorder/genetic disorder	6 (54.5)	
Duration of illness (months)		48.0 (58.7/8–180)
Child’s age at death		7.0 (3.9/2–15)
Months since death		55.0 (34.4/9–132)
Siblings		
Yes	10 (90.9)	
No	1 (9.1)	

*N*, number of participants; SD, standard deviation.

### Data collection

Data were collected through individual semi-structured interviews *via* a digital video platform, performed by the third author. Nine of the participants were interviewed independently, while one couple was interviewed together. The interviewer had not met any of the participants before the interviews. Before each interview, the researcher introduced herself and the aim and method of the interview. The interviews followed a semi-structured interview guide, where the main question was ‘Can you please tell me about the child you have lost and what happened?’ as outlined in Table 2.

**Table 2.** The semi-structured interview guide.

Interview question	Palliative stage	Possible themes for follow-up questions
Can you please tell me about the child you have lost and what happened?	Pre-palliative	Illness Time frame Information about the situation
Can you please share your experience of the municipal healthcare system’s care and support for the child and yourself?	Palliative	Hospital Home-based nursing/community PPC The child’s symptoms and distress Family support (siblings, parents, others) What worked/did not work
Can you tell me about what happened during the last days and hours of the child’s life – and what help and care you received from the healthcare system during this time?	Terminal phase	Who were involved? Symptom management Special challenges Competence of healthcare personnel
How did you experience the support from the healthcare system after the death of your child?	After death	
Based on your experience, what advice would you give to municipal healthcare services, about taking care of severely ill or dying children and their families? How could the services be improved?		Advice for professionals
Is there anything else you would like to add before we end the interview?		Express gratitude for participation

PPC, paediatric palliative care.

Each question was followed by additional questions to elaborate on the parents’ lived experiences. The interviews ranged in duration from 63 to 117 min. The participants provided information-rich data, whereby the sample provided coherent stories firmly grounded in the empirical data.^24^ All interviews were digitally recorded, and only the audio files were saved to protect the informants’ anonymity. The audio files were transcribed verbatim and anonymized, in a total of 255 pages, before further analysis.

### Data analysis

The data were analysed using systematic text condensation (STC), which is a four-stage method for thematic analysis of qualitative data and is well-suited for data collected within an interpretive descriptive research design.^24^ The analytic process is provided in Table 3.

**Table 3.** The analytic process using STC.

Step 1: Obtaining a total impression			
Process	Identified total impression		
a) The authors read the transcribed interviews separately and then b) discussed the total impressions to consensus	-Parents mainly interacted with the hospital, community services involved late -Children with multidimensional needs and regular medical crisis -Limited services and competence at the community level. Experience fighting the community system. -Parents under constant distress, with high care burden and medical responsibilities-feeling alone and exhausted -Siblings under the radar -‘We need flexible and holistic services from the community’. -Lack of grief support		
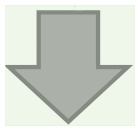			
Step 2: Identifying meaning units			
Process	Identified meaning units		
a) The authors coded the data separately and then b) discussed the codes to consensus within the codes	Meaning unit	Files	References
	Children’s complex needs	10	111
	Mainly interacted with the hospital – limited cooperation with the community	10	70
	Fighting the community system – all on one’s own initiative	10	71
	Communities’ limited services and competence	11	150
	Parents under distress – feeling left alone	11	135
	Lack of preparation for the terminal phase	11	66
	Lack of grief support	11	64
	Siblings’ situation 10	10	70
	Parents advice to communities	11	59
	*Files* *=* *number of informants who mentioned the meaning unit.* *References = total number of mentions of the meaning unit*.		
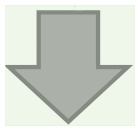			
Step 3: Abstracting the contents of the individual meaning unit			
Process	Abstracted contents/themes		
a) The authors analysed the contents separately and then b) held several discussions to achieve consensus including the context and the author’s preunderstanding	Five main themes: 1. We mainly interacted with the hospital 2. We did not get the help we needed 3. The child’s needs became increasingly complex 4. When the end came 5. Our family needed an ordinary life in an unordinary situation		
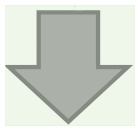			
Step 4: Summarizing the findings			
Process	Abstracted contents/themes		
a) The authors discussed the findings against the transcribed interviews and then: b) each author found direct statements to elucidate units of meaning and discussed these to consensus	The authors summarized the findings and presented direct statements within the abstracted contents.		

STC, systematic text condensing.

In the first stage, all authors read the transcriptions separately, before having a joint discussion to identify preliminary themes (step 1 in Table 3). The next stage involved rereading and coding the data separately, before having a new discussion to identify units of meaning to reach consensus on the units. The data were then reread and coded according to units of meaning using the NVivo software (QRS International, Denver, Colorado, USA) (step 2 in Table 3). The third step involved an analytical circle, where the authors discussed the units of meaning and classified them into groups and subgroups (step 3 in Table 3). In the fourth step, the identified themes and subthemes were validated against the transcribed interviews, whereby illustrative quotes were extracted and described in the text (step 4 in Table 3). However, STC is not a linear process; it allows the researchers to move between the steps in the process of interpretation.^24^ To facilitate reflexivity, each step was carefully discussed until consensus on all the interpretations. In this analytic process, we especially focused on how the context and the researchers’ preconceptions might have influenced the interpretations. Then, the findings were validated against the transcribed interviews to ensure that the informants’ expressed and intended meanings had been captured.

## Results

The analysis showed that the parents experienced the PPC in the community overall as limited and fragile, lacking flexibility, coordination and professional competence related to the children’s complex needs, with only a few exceptions. The parents’ experiences were captured in five main themes: (i) ‘Interaction with hospital and community services’, (ii) ‘Parents did not always get the help they needed’, (iii) ‘The child’s needs became increasingly complex’, (iv) ‘When the end came’ and (v) ‘The parents asked for an ordinary life in an unordinary situation’. Each of these themes was further elaborated by two subthemes as illustrated in Table 4.

**Table 4.** Overview of the study’s findings.

*Theme 1.* Interaction with the hospital and community services	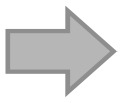	*Subtheme 1a.* Limited cooperation between the hospital and the community
		*Subtheme 1b.* Community involved late
*Theme 2.* Parents did not always get the help they needed	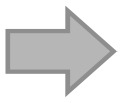	*Subtheme 2a.* Fighting the system
		*Subtheme 2b.* Lack of competence, coordination and flexibility
*Theme 3.* The child’s needs became increasingly complex	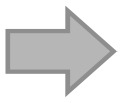	*Subtheme 3a.* Living with constant distress – feeling left alone
		*Subtheme 3b.* Siblings under the radar
*Theme 4.* When the end came	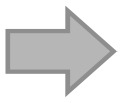	*Subtheme 4a.* Lack of preparation
		*Subtheme 4b.* Lack of bereavement support
*Theme 5.* The parents asked for an ordinary life in an unordinary situation	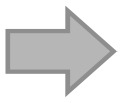	*Subtheme 5a.* Family focus and flexibility
		*Subtheme 5b.* Knowledgeable and coordinated services

### Theme 1: ‘Interaction with hospital and community services’

The first theme reflected the fact that the parents spent a lot of time at the hospital during their child’s illness. During the illness trajectory, the participants experienced several acute hospital readmissions. When they were at home, they often contacted the hospital for help or discussions. The parents described how they developed a trusting relationship with the professionals at the hospital, but such trust did not translate to the community:

We managed to create a unique cooperation (. . .) We got new routines; he always got admitted to his ward, so that if I was displeased with something, I could talk to his doctors, and we would find a solution. But that part, we didn’t manage to do the same with the community. (Informant 3)

This theme was further elaborated by two subthemes. The first subtheme, ‘Limited cooperation between the hospital and the community’, expressed how parents experienced that the hospital failed to establish contact with their local community and between the different community systems involved. Consequently, they experienced that the cooperation between the hospital and the community was limited, non-existent or challenging, for example when the community system did not act upon the hospital’s recommendations:

The doctors wrote medical certificates concerning his needs for help, but, as the community was in charge of distributing the services, they chose not to look at it. (Informant 4)

In contrast, some parents experienced cooperation with the community where they experienced being seen, their needs being met and the help coordinated:

We moved to another municipality, and we had a meeting with the allocation office, the child welfare, with (. . .) the GP (. . .), where everyone joining our support team were gathered. (. . .) And the best safety and support we could get (. . .) was knowing that if I called them and said I needed them, I would be heard. And I think this caused us to need less [help] than if I had been anxious . . . (Informant 8)

The second subtheme, ‘Community involved late’, underlined the fact that the community became involved at a late stage in the illness trajectory. As hospitals usually did not initiate cooperation with the community, the parents did not know whom to contact to establish support at home. Consequently, parents typically had to initiate contact themselves:

I think it would have been very important and very right if this meeting had been arranged without me initiating it. When a child is seriously ill, this meeting should be a routine, not something you demand. (Informant 1)

The informants stated that the community rarely reached out to provide services or to plan for the time ahead. However, some parents experienced that the community initiated early contact and arranged different services for the child and the family:

The community was involved early (. . .) collaborated with the kindergarten and . . . (. . .) and we communicated throughout the process (. . .). My parents became ‘support persons’ and they cared for him every third weekend. (. . .) She [the coordinator] made an individual plan with the community coordinator. And when I needed equipment, the question was always ‘what do you need?’ (Informant 11)

### Theme 2: ‘Parents did not always get the help they needed’

The second main theme outlined that parents generally experienced that they did not get the necessary help from the community system. Taking care of the child at home presented several challenges for the families, such as the following-up of medical treatments, adapting to changes in the child’s functioning, and being constantly on the watch for symptomatic changes. At the same time, the chores of daily family life were as demanding as ever:

We started complaining, and I told them [the community services]: ‘My husband is awake all night, watching [the ill child]. Then he goes to day-care, and my husband goes to work. This is not justifiable (. . .) it does not work’. In the end, we got night-time watchers, but we had far too little help. (Informant 4)

Overall, the parents reported that they did not know what kind of support the community system could provide, nor what rights they had as caregivers for a severely ill child. Parents mainly felt that their needs for support, to some or large degree, went unmet. These experiences were further elaborated by two subthemes.

The first subtheme, ‘Fighting the community system’, described how gaining access to services was expressed as a main challenge for the informants. Here, the parents stated that they had to fight to receive the help they needed, and that this took away precious time and energy that otherwise could have been spent with their child:

We asked [the municipality] at a very, very early stage. We realised that we could not handle this. (. . .) We asked the municipality, and this turned into a five year long battle between the hospital and the municipality about who was going to help us. (Informant 3)

In particular, parents perceived application processes regarding services as rigid and lengthy, or that community staff discouraged them from applying for the support they wanted. The informants stated that they continuously had to provide proof of their needs, or their parenting skills, in contact with municipal staff:

You must write applications with the proper language. You always have (. . .) to present the situation and yourself as a parent. I felt I was in an eternal audition in the community. (Informant 2)

In contrast, some parents reported that they had been surrounded by community systems that had been eager to offer support, and that support arrived swiftly and according to the parents’ preferences:

So, whenever we needed more help, whatever it was, it was just to call [name of coordinator]. Two days later it was arranged. (Informant 8)

The second subtheme, ‘Lack of coordination, competence and flexibility’, addressed the limitations in the services provided. The most requested home-based services were medical support, personal assistants, auxiliary aids, physical therapy, adaptations to enable school or kindergarten attendance and financial support for caregivers. Parents commonly requested help with medical care at home, but they found that competence in PPC within the home-based community nursing teams was close to non-existent:

It all seemed incredibly ill-prepared (. . .) They may have treated many dying elderly people (. . .) but they did not have competence regarding child palliation (. . .) I sensed an enormous insecurity (. . .) When they came, I had expected to be able to sit down for five minutes, knowing that someone else was taking the responsibility (. . .) I felt as if I had to stand by their side the entire time, watching them, and having to correct things they did. (Informant 9)

Parents who witnessed staff uncertainty or mistakes often felt distrust, which in several cases led to them refusing further help from home-based care providers or respite in community homes. Due to a lack of coordination, parents generally became responsible for conveying information and requests between services, and in some cases for carrying out urgent practical tasks, such as rushing to obtain auxiliary aids or medical equipment at different locations. Furthermore, participants largely experienced a lack of service flexibility regarding the time and place, and the amount of support, such as when medical procedures should be conducted, or flexibility and facilitation for the child to be able to attend school or kindergarten as much as possible.

Only some parents expressed opposite experiences, including effective coordination and flexible services:

They did a great job (. . .) and only five professionals were involved (. . .) and they had a service attitude. No problems were too big. (Informant 11)

### Theme 3: ‘The child’s needs became increasingly complex’

The third main theme described how the families’ situations increasingly worsened during the illness trajectory. The complexity of the child’s needs increased over time, including frequent hospitalizations, acute and invasive procedures, and new medications. All informants feared losing their child several times during the illness trajectory. This main theme was elaborated by two subthemes.

The first subtheme, ‘Living under constant distress’, illustrated the stressors in the families’ life situations. As the child’s care needs increased, the parental care burden expanded. Parents arranged meetings, cared for their child and siblings, administered medication, figured out how to get hold of necessary technical aids and coordinated the home care services. In addition, their personal networks were lost, and parents had to manage anticipatory grief in the family. This experience was one of constant distress and a feeling of overwhelming responsibility. The informants expressed how they felt powerless and helpless, and that they had been left on their own. One informant described this period as ‘a horrible nightmare that was actually real’. This situation resulted in a lack of sleep and the disruption of regular meals for the parents:

I couldn’t sleep or eat. It was really awful. Losing a child is one of the worst things that can happen to a human (. . .) It is so extremely exhausting and hard to be a parent. (Informant 1)

The second subtheme, ‘Siblings under the radar’, also elaborated on this distress. During the child’s illness, especially during hospitalization, those parents who had other children stated that they often had to deprioritize siblings. Siblings frequently had to wait while medical procedures took place, they did not get the help they needed with homework, some had to be home-schooled to reduce the risk of infections or had to stay with friends or extended family while their parents were at the hospital. The parents generally reported feelings of remorse regarding their other children:

He was 14 and got terrible care. He was basically left out in the cold. I have got such a guilty conscience about him. But you just cannot do it all (. . .) I couldn’t. I didn’t manage to do a good job regarding him. (Informant 1)

### Theme 4: ‘When the end came’

The fourth main theme focused on the children’s terminal phase. Parents mainly felt that neither they nor the community systems were prepared for this phase:

The plan was to go home on Monday. There was no cooperation with the community, no dialogue, nothing, even though we were going home with a terminally ill child. Nobody at the hospital had any plans for us, apart from telling us to call them [the hospital] if we needed anything. (Informant 2)

This theme was elaborated through two subthemes. The first subtheme, ‘Lack of preparation’, showed how parents did not feel properly prepared for when the end would come and how this would happen. The informants noticed their child’s health deteriorating over time. However, the parents did not experience that they were informed clearly about their child’s prognosis and took part in planning for the terminal phase. Only one mother expressed that she was able to prepare her child for death by reading books and answering the child’s questions:

We borrowed books about death to try to answer [the child’s questions]. (. . .) So, we had many good conversations, mainly in the car or in bed (. . .) He asked and dug. And I answered the best I could. And he wasn’t afraid to die. (Informant 11)

It was usually unclear when the transition from curative to palliative treatment took place. In general, the parents did not know what symptoms to expect during the terminal phase. The parents preferred a home death for their child, and most were granted this wish. Commonly, parents felt that the community healthcare systems were unprepared for the PPC task, and in some cases, parents returned home with terminally ill children without any form of community assistance. Regardless of the place of death, the parents were content with the circumstances of the children’s death and experienced it as being peaceful.

The second subtheme, ‘Lack of bereavement support’, described how the informants generally lacked professional support after their child’s death. The first days and weeks after their loss were experienced as hectic, often occupied by funeral preparations, memorial services and dealing with authorities. In general, the parents did not realize how tired they were, nor how heavily the burden of grief weighed upon them, until several weeks after the death:

I like to illustrate it with a picture, a metaphor. After the funeral, I was like an empty balloon that had been thrown away into a corner, without any air. That was me. Completely drained. (Informant 11)

Parents typically experienced bodily pains, tiredness, lack of concentration, sleeping problems and intrusive thoughts, and were not ready to go back to work for an extended period. All parents expressed a desire for bereavement support on behalf of themselves and their siblings. Some families were offered therapy or support groups by the hospital that had treated their child, while others searched for peer support groups or professional grief counselling by themselves:

Meanwhile, we parents were just left to ourselves. The municipality has never called us to ask if we needed anything or if there was a need for any kind of follow-up. We never heard from them again. (Informant 2)

Parents rarely received any bereavement support from the community. Siblings received limited support from the community healthcare nurses at their school, and their experiences of professional bereavement support varied greatly.

### Theme 5: ‘The parents asked for an ordinary life in an unordinary situation’

The final theme consisted of recommendations parents would like to give to communities regarding PPC. This includes positive experiences and thoughts of how the parents wished that their communities had supported them. Here, two subthemes emerged.

The first subtheme, ‘Family focus and flexibility’, underlined that the informants wished for a more holistic and family-focused approach from the community, together with an additional focus on the families’ psychosocial needs. The parents yearned for an ordinary family situation, including ordinary school attendance, work attendance and visits and where the family could be together and ‘make the days as good as possible’ (Informant 8). For this to be possible, they argued that there is a need for flexibility and adaptations in the community care system:

I wish we’d had a meeting with the community and talked about ‘How can we do this? How can we find good solutions for the entire family? What does this family need?’ For us, it was so important to be physically active, we had children that did sports, (their father) and I did sports. So, for us, it was so important to maintain this (. . .) You must figure out what is suitable for each family. That is so important. (Informant 3)

Some families received user-controlled personal assistance. This was highly appreciated as it allowed them to choose who was going to care for their child, as well as providing them with greater flexibility in their everyday life. Such a solution was also asked for by other parents and suggested by them as an important way in which to provide community PPC.

The second subtheme, ‘Knowledgeable and coordinated services’, emphasized the informants’ crucial need for coordinated and competent services. The parents expressed a wish for the community to reach out during an early stage of the illness trajectory, that there had been a contingency plan and that they had been offered information about what services were available:

Make offers, and then let us as families choose from them. Because to sit by the bed of your dying child and be expected to understand what the school can do for you (. . .) You aren’t there, that is not how it works. (Informant 8)

In general, the parents pointed to the need for improved accessibility to community services, and a functional information flow between community healthcare, hospital, school and kindergarten. Parents who had a designated municipality coordinator to contact and coordinate the services appreciated this greatly, while the rest expressed a wish to have such a coordinator. Furthermore, the parents underlined the importance of specific competence and skills among those involved in community-based PPC:

It is not about the personnel who were sent to my home, this is a question of management. I’m sure they did the best they could, and many times I felt their insecurity. They felt forced into a situation in which they did not feel comfortable. (Informant 9)

In general, the parents emphasized the importance of close collaboration between the professionals at the hospital and in the community, to develop competence within PPC at the community level.

## Discussion

To our knowledge, this is the first study to explore parents’ experiences of PPC in the community healthcare system in a Norwegian context. Here, several aspects of their experiences seem crucial for the development of community-based PPC.

One main finding was that the parents largely interacted with the hospital in the palliative phase. This may not be surprising, considering that both Norwegian practice and international research indicate that professionals at the specialist level possess the most paediatric competence and perform most PPC, even when the children stay at home.^1,2,7,26^ Aligning with previous research,^1,2,5,27^ the parents preferred to stay at home as much as possible. However, the results indicate that the hospitals seldom initiated cooperation with the community healthcare system, and, when included, this system was included at a late stage. As palliation is recommended to start at the time of the diagnosis,^1,2^ our findings may point to limited or lacking routines for early community inclusion at both hospital and community levels.

When the community system was included, the parents did not feel that they received sufficient support and necessary respite during the illness trajectory. Previous research suggests that efficient symptom management, psychosocial aspects of care, efficient care coordination and access to respite are the most important aspects of functional PPC.^5,15,18^ One upsetting finding was that many parents experienced having to fight against the community system for their needs to be met, instead of being given information about their rights and the help and support that was available. These findings highlight the need for structured initiation and coordination of services, to ensure equal support opportunities for all families. Overall, the parents were dissatisfied with the services provided and the community practitioners’ PPC competence. There seems to be a disparity between the complex needs of the families, whose wishes generally align with the aims of PPC,^3^ and the task-specific community health services. The provision of more holistic and family-oriented care, that also aims to include the psychosocial needs of the family, would be likely to require the active involvement of several community services in PPC, including the healthcare system, school and kindergarten, habitation services and allocation offices, as well as respite and access to auxiliary aids. This requires communication and cooperation across different organizations in Norwegian communities and between the healthcare levels. This is highly feasible by employing a dedicated and competent coordinator, who is a contact person for parents and responsible for gathering the different community and specialist service providers at regular meetings. An individual care plan must be created within this ‘responsibility’ group, and the allocation of the care plan tasks must be clarified.

The children’s needs became increasingly complex throughout the illness trajectory, whereby the parental responsibilities and distress increased. This aligns with previous research^10,28^ showing that caring for sick children at home can be physically and emotionally exhausting. Parental distress is to be expected when a child is terminally ill, as losing a child is one of the most stressful events people can experience.^29^ However, high-quality PPC has the potential to reduce the level of distress and increase the quality of life for the entire family.^11,14^ In addition, Smith and colleagues^19^ emphasize that the lack of respite in PPC, in line with our results, may be linked to increased parental distress. Furthermore, our findings indicate that the parents had to prioritize their ill child, resulting in siblings often being deprioritized and not receiving the support they needed. In Norway, children, as next of kin, have a legal right to receive proper support and follow-up.^30^ Our results indicate that both the community and specialist healthcare systems failed to follow-up on their responsibility in this area. The entire family system is affected by parental distress, the complex needs of the ill child, and the challenges experienced by the siblings. This underlines the importance of support for the entire family, as stated in the PPC definition.^3^

When the end came, most parents felt as if neither they nor the community support system were prepared for the terminal phase. One important principle of PPC is planning for the time ahead.^1^ A highly concerning finding was that some parents were discharged from the hospital during the terminal phase without any plans or community support. One could argue that this provides an example of a support system that failed to support families in accordance with the goals of PPC during an extremely vulnerable phase. The parents called for honest communication about their child’s prognosis, as well as information about likely future symptom development. The PPC population is a small group characterized by unclear prognoses and unpredictable illness trajectories.^1^ This may be a factor that complicates clear communication, as professionals may struggle to predict what lies ahead. As a lack of predictability is linked to distress,^31^ however, it seems that professionals should strive for clear communication to provide optimal PPC.

As most support was directed at the ill child, our findings indicate that the support from the community system stopped abruptly when the child died. PPC includes the psychosocial needs of the family after the child’s death.^3^ The lack of bereavement support, therefore, seems to constitute a clear deficiency in the communities’ support for the families. Several studies underline the importance of both high-quality PPC and bereavement support, as families who lose a child are at risk of developing complex grief reactions.^12^ Furthermore, low-quality PPC impairs parents’ bereavement outcomes,^11,14^ and lack of psychosocial support in grief increases the likelihood of complicated grief reactions.^32^

Based on their experiences, the parents outlined several implications for clinical practice. In alignment with previous research,^15^ the parents stressed a need for care coordination. Specific areas for improvement appeared to concern information about potential support services, easy access to services, regular collaboration meetings and a designated community coordinator to help parents coordinate the services at both the community level and the specialist healthcare level. Another essential factor was PPC competence within the community healthcare services. Perceived lack of competence affects the relationship between parents and healthcare providers and may also affect parental well-being.^8^

Our findings also highlight the potential for improved community PPC through better cooperation between the hospital and the community, improved services at the community level, greater focus on preparing for the terminal phase, better routines for grief support and holistic and family-oriented care. The findings stress that families want to stay at home, but also show how the families differ in needs and habits, which indicates the importance of flexible and individually adjusted PPC. It can be argued that it can be difficult for small communities that rarely have children in need of PCC to build competence in this field. However, as PPC is a law-based service in Norway, all communities are obliged to provide PPC in close collaboration with hospitals.^2^ The implementation of hospital-based paediatric palliative teams and home-hospitals in Norway^2^ is therefore an important and promising development for these families to be able to stay at home as much as possible, but our results may indicate that this work still needs to be implemented in cooperation with the community healthcare services. However, as the ill children’s needs are complex and include the entire family’s situation, home-based PPC cannot solely be built on support from the specialist healthcare level that, in Norway, are only available in the daytime (9:00–15:00 a.m.). There is also no children’s hospice in Norway. Consequently, our results indicate the importance of developing a functional model for PPC in community healthcare, including PPC competence, structures for cooperation between the specialist and community healthcare level, care coordination at both the community level and the specialist level and the inclusion of a family perspective both during and after death.

### Strengths and limitations

The strengths of this study include the novel focus on parents’ own experiences of community PPC, as well as the fact that the informants had lost children with different diagnoses and with greatly varying duration of the illness trajectories. Parents of different family systems (only/multiple children, two/single parents) were included, and participants were recruited on a national level representing rural and urban geographical communities. All these are important characteristics of qualitative research. Although only 11 informants were included, these informants provided rich data material. The analysis was transparent, and the findings were consistent, suggesting that the findings are credible and firmly grounded in empirical data. Letting different informant’s voices be heard *via* direct quotes supports the study’s authenticity. The critical discussions between the authors during the research process, the intercoder agreement in the analyses, and the discussion, support the researchers’ critical appraisal. To address self-critic (integrity), this study also has some limitations. A limitation of the study may be that we only recruited one father, whereby a more even gender balance might have rendered other perspectives. Other limitations may be that PPC in general was introduced late in the illness trajectory, and that the parents shared their stories in retrospect. On average, it was nearly 5 years since the passing of the child. This may have influenced the parents’ retrospective story. Furthermore, we did not ask for the informants’ ethnicity, religious or sexual orientation. Thus, we were unable to describe or highlight potentially distinctive experiences based on demographic factors. This may have hampered the variety of representation in the sample.

## Conclusion

The findings from this study show that the parents mainly interacted with the hospital, and they experienced the paediatric palliation in the community as being limited and fragile, and lacking flexibility, coordination and professional competence related to the children’s complex needs, as well as a lacking of family focus. The results indicate that further development is needed to provide PPC in community settings that align with WHO standards. As most PPC competence is gathered at the specialist healthcare services, knowledge exchange and close cooperation between the hospital and community, with common care plans and unified care for the family, seem to be crucial for optimal home-based PPC. There is a need for increased focus on the psychosocial needs of parents and siblings. It would be beneficial for the families if the community organized its services in a manner that ensures availability, predictability and clear roles and responsibilities, where the holistic needs of the entire family are considered. As community PPC is a research field that is still in its early days, further research and the development of evidence based-PPC models are necessary to promote the evolution of the field.

## Declarations

**Ethical approval and consent to participate:** This study project is part of a larger research project related to municipal paediatric palliation, led by professor MAH. The project was assessed by the Regional Committee for Ethics in Medical and Health Research (REK) and concluded to be outside the Norwegian Act of health research (Helseforskningsloven) (reference number: 435941). The project was approved by the Norwegian Data Protection Authority (reference number 270888) and registered in the institutional system for research projects (RETTE: ID number F2082). Eligible participants received verbal and written information about the study, including that their participation was voluntary, and that they could withdraw at any time. Participants who consented to participate gave their telephone number or e-mail address to the researcher. The project manager then contacted the informants and provided them with additional information about the study’s aim and the interview process. Participants’ consents were verbally repeated on tape prior to each interview. As the interview topic was of strong emotional significance, and involved recall of potentially traumatic events, the participants were given the opportunity to contact the interviewer post-interview for follow-up. No participants withdrew from the interview situation, nor asked for follow-up. The researchers followed established guidelines for preserving anonymity and the safe handling of the data based on the institutional routines and the Helsinki declaration.

**Consent for publication:** All participants got written and verbal information about the publication of data. All consented.

**Availability of data and materials:** The data that support the findings of this study are not publicly available due to privacy or ethical restrictions. The data are available on reasonable request from the professor MAH (e-mail: may.hauken@uib.no).
